# Optimization of Medium Composition for the Production of Neomycin by *Streptomyces fradiae* NCIM 2418 in Solid State Fermentation

**DOI:** 10.1155/2014/674286

**Published:** 2014-06-09

**Authors:** B. M. Vastrad, S. E. Neelagund

**Affiliations:** ^1^Department of Pharmaceutical Biotechnology, S. E. T's College of Pharmacy, Dharwad, Karnataka 580 001, India; ^2^Department of PG Studies and Research in Biochemistry, Jnana Sahayadri Kuvempu University, Shankarghatta, Karnataka 577 451, India

## Abstract

Neomycin production of *Streptomyces fradiae* NCIM 2418 was optimized by using response surface methodology (RSM), which is powerful mathematical approach comprehensively applied in the optimization of solid state fermentation processes. In the first step of optimization, with Placket-Burman design, ammonium chloride, sodium nitrate, L-histidine, and ammonium nitrate were established to be the crucial nutritional factors affecting neomycin production significantly. In the second step, a 2^4^ full factorial central composite design and RSM were applied to determine the optimal concentration of significant variable. A second-order polynomial was determined by the multiple regression analysis of the experimental data. The optimum values for the important nutrients for the maximum were obtained as follows: ammonium chloride 2.00%, sodium nitrate 1.50%, L-histidine 0.250%, and ammonium nitrate 0.250% with a predicted value of maximum neomycin production of 20,000 g kg^−1^ dry coconut oil cake. Under the optimal condition, the practical neomycin production was 19,642 g kg^−1^ dry coconut oil cake. The determination coefficient (*R* ^2^) was 0.9232, which ensures an acceptable admissibility of the model.

## 1. Introduction

Neomycin is a topical antibacterial antibiotic with low toxicity. This antibiotic is employed for treating a variety of bacterial infections including diseases of skin, wound injury, and tuberculosis. This antibiotic is also used in veterinary practice, in storage tanks of petroleum fuels where it prevents the formation of sludge, and in rubber tree plantations where it increases the flow of latex by preventing bacterial infection of tapping wounds [1]. Neomycin is a bacteriostatic compound active against Gram-positive, Gram-negative, and acid-fast bacteria [2]. Neomycin was first isolated from* Streptomyces fradiae* [2]. Extensive literature is available on neomycin production by* Actinomycetes* spp. [3]; however,* Streptomyces fradiae* was established to be the best strain for neomycin production [4, 5].

Traditionally, neomycin has been produced by submerged fermentation (SmF). In recent years, however, the solid-state fermentation (SSF) processes have been increasingly applied for the production of this antibiotic [6]. SSF compared to SmF is more simple, requires lower capital, has superior productivity, reduced energy requirement, simpler fermentation media, and absence of rigorous control of fermentation parameters, uses less water and produces lower wastewater, has easier control of bacterial contamination, and requires low cost for downstream processing [7, 8]. In the SSF process, the solid substrate not only supplies the nutrients to the culture, but also serves as harborage for the production strains. The moisture content of the medium changes during fermentation as a result of evaporation and metabolic activities and thus optimum moisture level of the substrate is therefore most important [9, 10].

One of the largest oil cakes abundantly produced in India is coconut oil cake from the edible oil extraction industries. Coconut oil cake is dried and marketed as a component of animal feed, but since the selling price of the products is low and the evaporation of water from this waste consumes large amounts of heat, the production of dried coconut oil cake feed is valuable only as a waste disposal method, with marginal economic benefits [11]. Edible oil cakes have a high nutritional value, especially having protein content ranging from 15% to 50% (http://www.seaofindia.com/). Their composition varies depending on their variety, growing condition, and extraction methods [12]. Several processes and products have been reported that utilize coconut oil cake as a raw material. These include products of fermentation were different enzymes such as *α*-amylase, glucoamylase, inulinase, and phytase [10, 13–16].

Medium optimization was approached either classically [6] from two factorial (Placket-Burman Design) to multifactorial design (response surface methodology) [17], are accessible for overcoming such limitations. In the multifactorial design, the experiments involved are 2^*n*^, where* n* is the number of nutritional factors. Optimization of solid fermentation media is generally done at a two-step level, where initially the effects of the nutrients on product formation are screened and a few parameters are selected and optimized [18].

The optimal design of fermentation media is a considerable aspect to be sensible in the advancement of solid state fermentation processes. In the particular case of antibiotics, the interaction between growth metabolism and product secretion is critically affected by growth limiting nutrient concentrations. Designs of experimental techniques are very beneficial tools for this purpose, as they can accommodate statistical models with a relatively small number of experiments. From these models, the relative effect of various factors studied can be determined and their optimal concentration measured for a given target such as maximal antibiotic production.

The present work was directed at optimization of medium components, which have been initiated to play very capital role in decorating the neomycin production, with the accommodate of Placket Burman design and central composite design. In the introductory step of optimization, four carbon sources, four amino acids, four minerals, and four inorganic nitrogen sources were evaluated to arrange optimal carbon and inorganic sources, amino acids, and minerals. Then, a Placket-Burman design was exploited to describe which components of the media had significant effects on neomycin production; subsequently, a central composite design was employed to optimize the nutritional factors, which had significantly altered neomycin production. The results were analyzed by response surface analysis.

## 2. Materials and Methods

### 2.1. Organisms

*Streptomyces fradiae* NCIM 2418 was obtained from the National Collection of Industrial Microorganisms, Pune, India.

### 2.2. Culture Maintenance

*Streptomyces fradiae* NCIM 2418 was maintained on agar slants containing (g/L): glucose 4, yeast extract 5, peptone 10. The organism was subcultured every month and preserved at 4 ± 1°C.

### 2.3. Seed Culture Medium

The composition of the medium used for the development of seed culture was the following (g/L): soluble starch 20, tryptone soy broth 20, yeast extract 3, CaCO_3 _3, K_2_HPO_4_ 1, and MgSO_4_·7H_2_O 0.025. 500 mL Erlenmeyer flasks containing 100 mL of inoculum medium were autoclaved at 121°C for 20 min. The initial pH of the media was adjusted to 7.2 before sterilization. The seed culture medium was inoculated with 1 mL of spore's suspension (in sterile distilled water) containing 18 × 10^6^ spores from a 9-day slant. The culture was incubated for 4 days on a rotatory shaker at 160 rev per min and at 30°C. The age of the organism in the slant growth, seed age, and inoculum levels were maintained as described previously [5].

### 2.4. Coconut Oil Cake

Coconut oil cake was obtained from Kollam, Kerala, India, and was chopped by a chopper into small pieces, dried, and ground in a hammer mill. These ground materials were then separated by sieves into particles of desired size (moderate corse size) and were used in media. Dry weight and moisture content of the substrates were determined gravimetrically after drying the samples at 60°C.

### 2.5. Neomycin Production by Solid-State Fermentation (SSF) and Antibiotic Extraction

Neomycin production was carried out using coconut oil cake as the basic solid substrate unless otherwise stated.* Streptomyces fradiae* NCIM 2418 was grown in 500 mL Erlenmeyer flasks containing 10 g of each substrate and 0.2 g yeast extract, and moisture content was 45%. The initial pH of the growth media was pH 7.2 before sterilization. Both initial pH and moisture content were measured in preliminary experiments. All flasks were sterilized at 121°C for 20 min. To each flask, 2.0 mL of spore suspension was inoculated. The cultures were incubated statically at 30°C for 8 days.

After suitable periods of growth time, the neomycin was extracted from the fermented solid medium with 10-fold phosphate buffer by shaking (200 rpm) at 30°C on a magnetic shaker for 30 min. The suspended materials and fungal biomass were separated by centrifugation (10000 ×g for 15 min) and the clarified supernatant was used as the source of crude antibiotics.

### 2.6. Estimation of Neomycin by HPLC

Neomycin was estimated by HPLC-ELSD (evaporative light scattering detection) method reported by Nikolaos and Michael [19]. Walters HPLC system fitted with a Waters ODS-2 C_18_ Spherisorb column at evaporation temperature of 45°C was used. The optimized mobile phase was water-acetone (50 : 50), containing 11.6 mM heptafluorobutyric acid, in an isocratic mode at a rate of 1.0 mL/min. Neomycin was eluted at 4.9 min, with asymmetry factor 1.3. Logarithmic calibration curve was obtained from 2 to 50 *μ*g/mL (*r* > 0.9997). Limit of detection (LOD) was 0.6 *μ*g/mL and R.S.D. = 1.7% (*n* = 3, 3.3 *μ*g/mL). Concentration of neomycin was expressed in g kg^−1^of substrate.

### 2.7. Experimental Design and Data Analysis

#### 2.7.1. Determination of Optimal Minerals, Amino Acids, Carbon and Inorganic Nitrogen Sources

**Figure 1:**
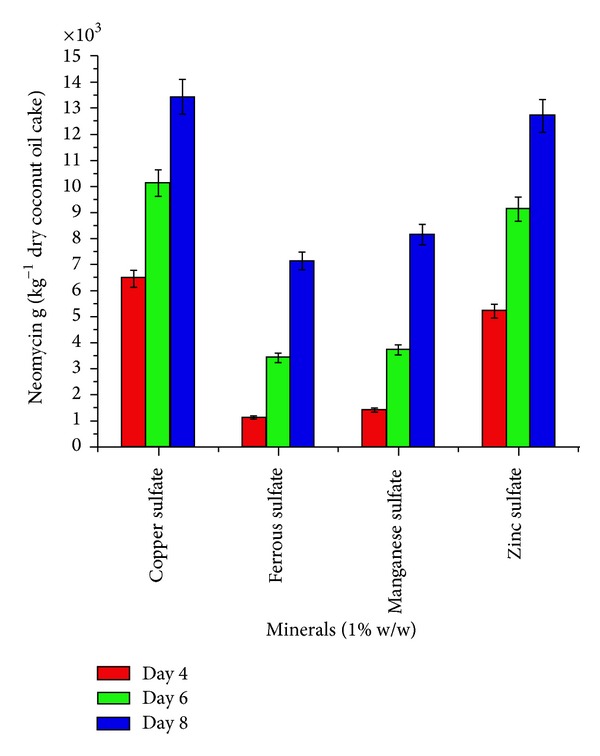
Effect of minerals (1% w/w) on neomycin production by* Streptomyces fradiae *NCIM 2418 using coconut oil cake under solid-state fermentation.

**Figure 2:**
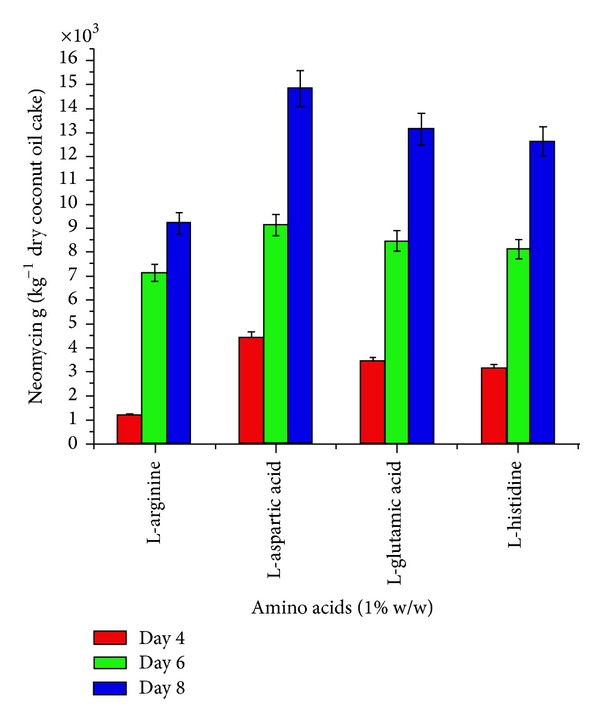
Effect of amino acids (1% w/w) on neomycin production by* Streptomyces fradiae *NCIM 2418 using coconut oil cake under solid-state fermentation.

**Figure 3:**
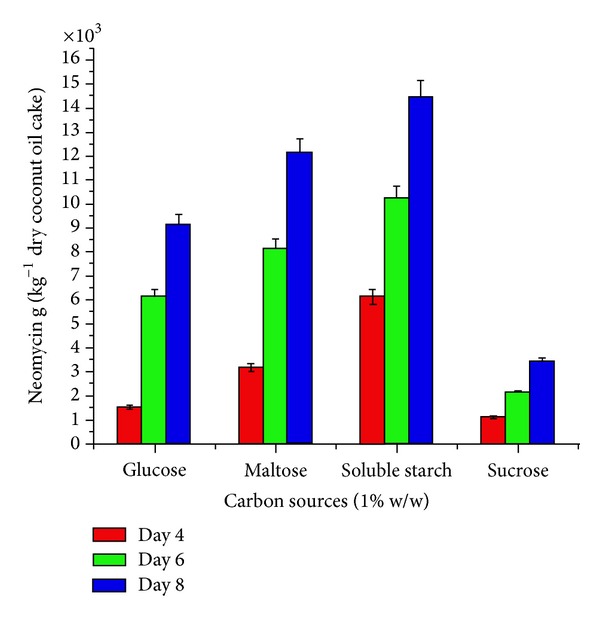
Effect of carbon sources (1% w/w) on neomycin production by* Streptomyces fradiae *NCIM 2418 using coconut oil cake under solid-state fermentation.

**Figure 4:**
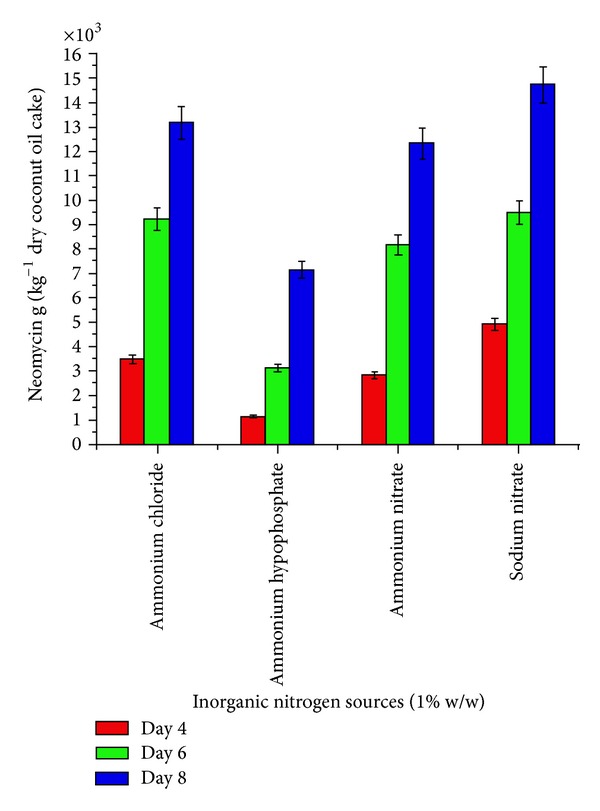
Effect of inorganic nitrogen sources (1% w/w) on neomycin production by* Streptomyces fradiae *NCIM 2418 using coconut oil cake under solid-state fermentation.

To select the appropriate minerals, amino acids, carbon, and inorganic nitrogen source, in introductory step of optimization, four minerals (zinc sulphate, manganese sulphate, ferrous sulphate, and copper sulphate), four amino acids (L-histidine, L-glutamic acid, L-asparatic acid, and L-arginine), four carbon sources (sucrose, soluble starch, maltose, and glucose) and four inorganic nitrogen sources (sodium nitrate, ammonium nitrate, ammonium hypophoshate, and ammonium chloride) were evaluated (Figures 1–4). These nutrients were, respectively, added in to the flask with coconut oil cake as a basal medium. Initial moisture content of the media was familiarized to 40%. After sterilization and cooling to ambient temperature, 10 mL inoculum was inoculated in the flasks. The final moisture then was well kept at 140%. The neomycin production of* Streptomyces fradiae *was measured at intervals of day 4, day 6, and day 8 of incubation at 30°C.

#### 2.7.2. Placket-Burman Design

In multifarious cases, there are an ample number of nutritional factors, which require to be identified for their consideration to the conditional variable of attraction. The most glandular advance would be to very those nutritional factors in a central composite design, that is, to attempt all feasible combinations of settings. Full factorial design requires 2^*N*^ (*N* denotes number of nutritional factors) experiments. This would achieve good optimization condition, barring that the account of essential runs in the experiments (observations) will amplify geometrically. In our case, eleven variables to be examined, it requires 2^11^ (2048) experiments, which is very ample and time consuming.

Placket-Burman design is a very beneficial tool used to screen “*n*” variables in just “*n* + 1” number of experiments [18, 20, 21]. There will be an enormous cut-down in total experiments. In this part, the preferred minerals (copper sulphate and zinc sulphate), amino acids (L-histidine, L-glutamic acid, and L-asparatic acid), carbon (soluble starch, maltose, and glucose), and inorganic nitrogen sources (sodium nitrate, ammonium nitrate, and ammonium chloride) were optimized (Table 1). The design matrix (Table 2) was developed using an SAS package (trial version 9.1).

Each independent nutritional factor was set at two level, that is, high and low level (Table 1). The high level of each independent nutritional factor was set far-adequately from the low level to ascertain which nutrients of the media have influential effect on the neomycin production.

#### 2.7.3. Central Composite Design

The CCD is one of the response surface methodologies (RSM) [22]. After the identification of the nutrients touching the neomycin production significantly, a CCD was adopted to optimize the appreciable nutrients (ammonium chloride, sodium nitrate, L-histidine, and ammonium nitrate), which were preferred through Placket-Burman design.

A 2^4^ full factorial central composite experimental design with 10-star points (*α* = 2.0), six replicates at center points, and consequent in a total of 30 experiments was used to analyse the four preferred nutrients of the medium for the neomycin production of* Streptomyces fradiae *NCIM 2418 by SSF. The experiment was designed by SAS package, trial version 9.1. The various levels of variables and central composite design matrix were conferred in Tables 3 and 4. The experiments were performed in duplicate and the mean values are taken for analysis.

A second-order polynomial, (1), which admits all interaction terms, was used to calculate the predicted response:

(1) $$\begin{array}{c} Y=\beta_{0}+\sum \beta_{i}x_{i}+\sum \beta_{ii}x_{i}^{2}+\sum \beta_{ij}x_{i}x_{j}, \end{array}$$

where *Y* represents response (dependent) variable, *β*_*o*_ is the intercept coefficient, *β*_*i*_ is coefficient of the linear effect, *β*_*ii*_ is the coefficient of the quadratic effect, and *β*_*ij*_ is the coefficient of the interaction effect, Where *x*_*i*_ and *x*_*j*_ express the coded level of variables *X*_*i*_ and *X*_*j*_ investigated in experiments.

The variable *X*_*i*_ was coded as *x*_*i*_ according to the following:

(2) $$\begin{array}{c} x_{i}=\frac{X_{i}-X_{0}}{\Delta X_{i}}, \end{array}$$

where *x*_*i*_ is (dimensionless) coded value of the variable *X*_*i*_, *X*_0_ is the actual value of *X*_*i*_ at the center point (zero) level, and the Δ*X*_*i*_ is the step change value.

Media containing 10 g coconut oil cake as a basal medium from same batch was dispensed into a 500 mL Erlenmeyer flask and supplemented with the nutrients for optimization. The neomycin production of* Streptomyces fradiae *NCIM 2418 was verbalized in g kg^−1^ dry coconut oil cake. An SAS package, trial version 9.1, was used for multiple regression analysis of the experimental data obtained.

The* F*-test was occupied to calculate the statistical significance of the quadratic polynomial. The multiple coefficient of correlation *R* and the determination coefficient of correlation *R* ^2^ were calculated to estimate the production of the regression equation.

## 3. Results

### 3.1. Determination of Optimal Minerals, Amino Acids, Carbon, and Inorganic Nitrogen Sources

In the introductory step of optimization, the preferred nutrients were added to coconut oil cake separately. The complementary nutrient really advances concentration of neomycin of* Streptomyces fradiae *NCIM 2418.

The effect of supplementation with various minerals, amino acids, carbon, and inorganic nitrogen sources on neomycin production by* Streptomyces fradiae *NCIM 2418 is shown in (Figures 1–4). Minerals, amino acids, carbon, and inorganic nitrogen sources in the basal medium were at a level of 1% (w/w).

The effects of supplementation with minerals were studied at a level of 1% w/w and were generally found to have a moderate effect on the production of neomycin (Figure 1). Copper sulphate and zinc sulphate supplementation showed the highest production of neomycin at 13423 and 12687 g kg^−1^ dry coconut oil cake at eighth day of incubation.

The effect of the supplementation of organic nitrogen was studied by adding various amino acids at a concentration of 1% w/w. Results indicated that they almost completely stimulated the production of neomycin at eighth day of incubation. L-arginine did not affect neomycin yield (Figure 2). Almost all the carbon sources tested increased the neomycin production at eighth day of incubation. Sucrose reduced the production of neomycin (Figure 3).

Supplementation with various inorganic nitrogen sources gave a significantly higher neomycin yield at day 8 of incubation period. The addition of sodium nitrate, ammonium chloride, and ammonium nitrate at 1% w/w showed the highest yield of 14679, 13138, and 12314 g kg^−1^ dry coconut oil cake at eighth day of incubation (Figure 4).

Based on the above experiments, soluble starch, maltose, glucose, copper sulphate, zinc sulphate, L-asparatic acid, L-glutamic acid, L-histidine, sodium nitrate, ammonium chloride, and ammonium nitrate were selected for statistical optimization.

### 3.2. Evaluation of the Nutritional Factors Affecting Neomycin Productivity by Plackett-Burman Design

**Figure 5:**
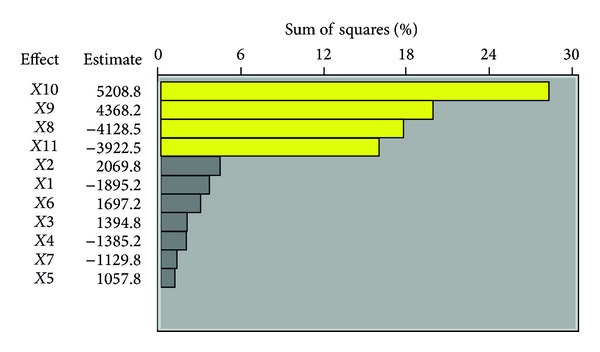
Pareto chart rationalizing the estimate of each variable on the neomycin yield (g kg^−1^) produced by* Streptomyces fradiae *NCIM 2418.

Eleven different nutritional factors including soluble starch, maltose, glucose, copper sulphate, zinc sulphate, L-asparatic acid, L-glutamic acid, L-histidine, sodium nitrate, ammonium chloride, and ammonium nitrate were sheltered for their effect on neomycin production using the Plackett-Burman design. The independent variables examined and their settings are shown in Table 1. The design plan and the averages of neomycin production for the different trials are given in g kg^−1^ dry coconut oil cake and shown in Table 2. The main effect of each variable was estimated as the difference between both averages of measurements made at the high level (+1) and at the low level (−1) of that nutritional factor. The data in Table 2 show a broad change from 2345 to 17835 g kg^−1^ dry coconut oil cake of neomycin production. This change reflects the account of medium optimization to accomplish higher productivity. The analysis of the data from the Plackett-Burman experiments involved a first-order (main effects) model. The main effects of the nutritional examined factors on the neomycin production were measured and conferred graphically in Figure 5.

On the basis of the analysis of the estimate of the eleven variables after day 8 of incubation, ammonium chloride and sodium nitrate showed large positive effect on neomycin production. L-histidine and ammonium nitrate showed large negative effect on neomycin production. Soluble starch, glucose, sucrose, maltose, zinc sulphate, manganese sulphate, ferrous sulphate, copper sulphate, L-arginine, L-asparatic acid, and L-glutamic acid have a slight effect on neomycin productivity. Figure 5 shows the ranking of nutritional factor estimate in a Pareto chart. The Pareto chart displays the magnitude of each nutritional factor estimate and it is a convenient way to view the results of a Plackett-Burman design.

### 3.3. Central Composite Design and Response Surface Method

According to Plackett-Burman design experiment, four significant variables* X*10 (ammonium chloride),* X*9 (sodium nitrate),* X*8 (L-histidine), and* X*11 (ammonium nitrate) were preferred for advance optimization using central composite design RSM to calculate the several optimal values. The coded values of the variables at various levels in central composite design (RSM) analysis were given in Table 3.

Response results shown in Table 4 were analyzed using SAS package, trial version 9.1, software. The* t*-test and* P* values were used to determine the effect of each nutritional factor on neomycin production (Table 6). Ammonium chloride, sodium nitrate, L-histidine, and ammonium nitrate and the interaction of the four preferred variables had a significant effect on neomycin yield (*P* < 0.05), as well as the quadratic terms of ammonium chloride, sodium nitrate, L-histidine, and ammonium nitrate. The fit of the model was checked by the coefficient of determination* R* ^2^, which was measured to be 0.9232, indicating that 92.32% of the changeability in the response can be explained by the model. The value of adjusted determination coefficient (*R*_Adj_^2^ = 0.8287) was also high enough to denote the significance of the model. The model can be shown as follows:

(3) Y(g kg−1)=38034.31+336.2×(BLOCK="1")−  5604.3×(BLOCK="2")−X10  999.87  +  X9  3278.04−X8  2514.37  +  X11  413.95+X10×X10  95.90  +  X10×X9  438.31+X10×X8  2550.93  −  X10×X11  2645.31−X9×X9  1563.46  +  X9×X8  2163.43+X9×X11  1436.93  −  X8×X8  428.96+X8×X11  1997.06    +  X11×X11  125.03,

where* Y* is the response, that is, neomycin concentration, and* X*10,* X*9,* X*8, and* X*11 are the coded values of ammonium chloride, sodium nitrate, L-histidine, and ammonium nitrate, respectively.

The significance of each estimate was determined by *P* values which are listed in Table 5. Condition for neomycin production had significant model because the value of *P* > *F* for the model was less than 0.05. Among the model terms, the linear effects of sodium nitrate and L-histidine were more significant than other nutritional factors. These suggest that the concentrations of sodium nitrate and L-histidine were a direct relationship with the neomycin production. The estimate for quadratic effect of sodium nitrate was more significant neomycin production. The interaction between ammonium chloride and L-histidine, ammonium chloride and ammonium nitrate, sodium nitrate and L-histidine, and L-histidine and ammonium nitrate had significant influence on neomycin production.

**Figure 6:**
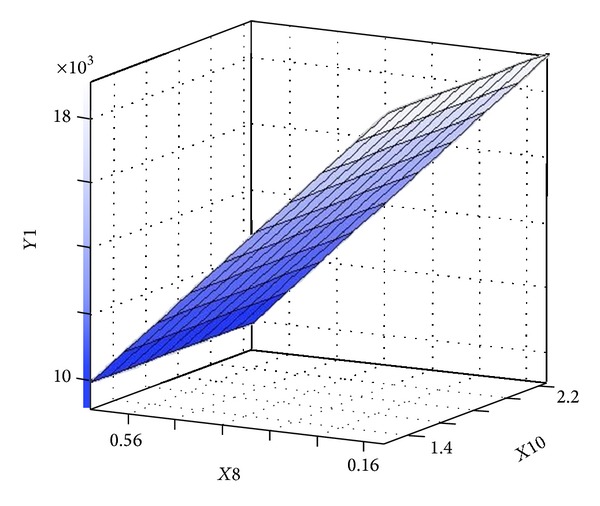
Three-dimensional surface plot of neomycin production as function of ammonium chloride (*X*10) and L-histidine (*X*8) (ammonium nitrate and sodium nitrate were kept at zero level).

**Figure 7:**
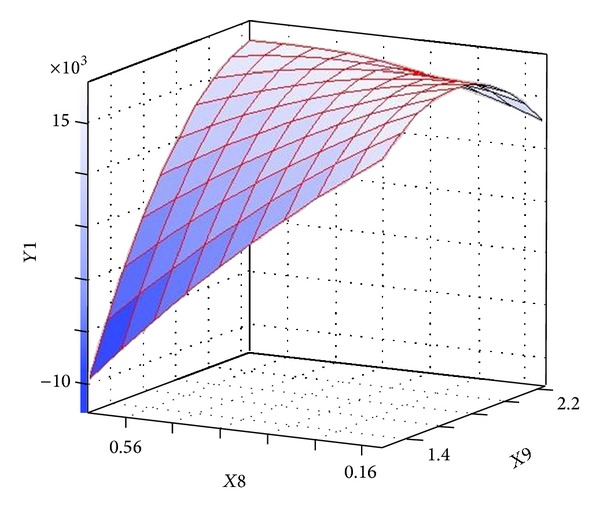
Three-dimensional surface plot of neomycin production as function of sodium nitrate (*X*9) and L-histidine (*X*10) (ammonium nitrate and ammonium chloride were kept at zero level).

**Figure 8:**
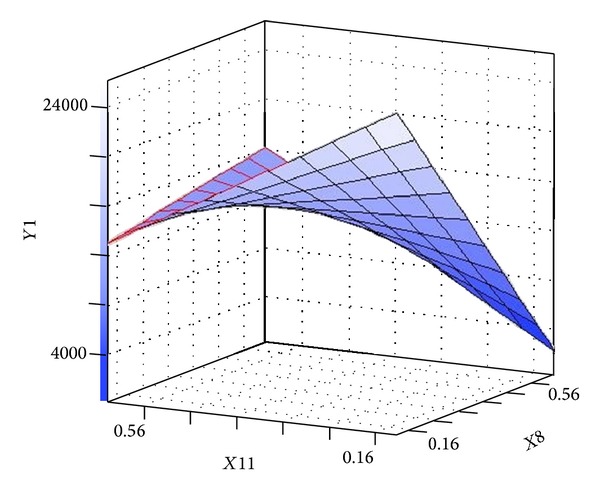
Three-dimensional surface plot of neomycin production as function of L-histidine (*X*8) and ammonium nitrate (*X*11) (sodium nitrate and ammonium chloride were kept at zero level).

**Figure 9:**
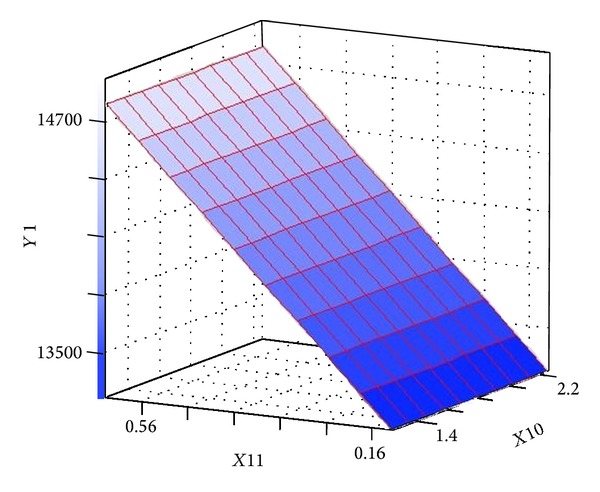
Three-dimensional surface plot of neomycin production as function of ammonium chloride (*X*10) and ammonium nitrate (*X*11) (L-histidine and sodium nitrate were kept at zero level).

Table 6 presents Fisher's* F*-test of analysis of variance (ANOVA), which also proves that this regression was statistically significant (*P* < 0.0001) at 95% of confidence level. Generally, the measured* F* value should be several times greater than tabulated* F* value if the model is admirable prediction of experimental results and the estimated nutritional factors effects are actual. In this case, the ANOVA of the regression model demonstrates that the model is highly significant, as is observable from the measured* F* value (=9.769752) and a very low probability value (*P* > *F* = 0.0001). Consequently, these results indicate a good adequacy in the models for neomycin yield. In order to achieve an advanced appreciation of the effects of the variables on the production of neomycin, the predicted model was presented as 3D response surface graphs (Figures 6–9).

The 3D surface plots of the neomycin production with respect to the concentrations of ammonium chloride and L-histidine are shown in Figure 6. The neomycin production increased with the increase concentration of ammonium chloride and L-histidine when sodium nitrate and ammonium nitrate were held at zero level.  Figure 7 showed effect of sodium nitrate concentration and L-histidine concentration on neomycin production when ammonium chloride and ammonium nitrate were held at zero level. It can be observed that an increase of neomycin production with increase in concentration of L-histidine and further increase in concentration leads to decrease neomycin production and increase in concentration of sodium nitrate with increases the neomycin production and further increase in concentration leads to decrease neomycin production. The three-dimensional response surfaces at various concentration of L-histidine and ammonium nitrate concentration were plotted in Figure 8. From the graph it was observed that the content of neomycin increases sharply when the concentration of L-histidine decreases below 0.5% (w/w). However the content of neomycin increased with the concentration of ammonium nitrate decreasing when ammonium chloride and sodium nitrate were held at zero level. Presenting experimental results in the form of three-dimensional surface plot, Figure 9 shows that higher concentrations of ammonium chloride stimulate the neomycin production and lower concentration of L-histidine support relatively high levels of neomycin yield when ammonium nitrate and sodium nitrate were held at zero level.

The predicted optimum levels of the tried variables, namely, ammonium chloride, sodium nitrate, L-histidine, and ammonium nitrate, were obtained by applying regression analysis of (3) by using SAS package, trial version 9.1. The optimal levels were as follows: ammonium chloride = 2.00% (w/w), sodium nitrate 1.50% (w/w), L-histidine 0.250%(w/w), and ammonium nitrate 0.250% (w/w) with corresponding response* Y* = 20,000 g kg^−1^ dry coconut oil cake. Verification of the predicted values was guided by using optimal concentration of nutrients in fermentation experiments. The practical comparable response was 19642 g kg^−1^ dry coconut oil cake. This result verified the weight and effectiveness of this model.

## 4. Discussions

The renewed interest in producing antibiotics from various production strains has spawned increased interest in solid state fermentation. Previous results indicated that optimal fermentation conditions in solid substrate cultivation must be selected experimentally for each organism/substrate combination [23]. Some general conclusions can be drawn in addition to the specific recommendations for cultivating* Streptomyces fradiae *NCIM 2418 on coconut oil cake.

Mahalaxmi et al. [24] reported that the solid substrate composition plays a vital role in the production of the antibiotics. In the present study the neomycin produced by the* Streptomyces fradiae *NCIM 2418 shows much higher than the literature reports. The optimal conditions for the production of neomycin by* Streptomyces marinensis* NUV-5 were determined in earlier work [1, 6] in which a dextrin, raspberry seed powder, and concentrated mineral (1% and 10% w/w) were used as nutrients. Production strain* Streptomyces fradiae* is a well-known neomycin production strain [4, 5, 25] and, therefore, enough of nutrients are required for cell growth and metabolism. Keeping this in view, several nutrients were screened (introductory step of optimization) for neomycin production. The data indicated that neomycin production pattern varied with the coconut oil cake.

The formal method (one factor at a time) for medium optimization is time consuming and cannot depict the combined effect of multiple processes parameters involved [26]. The Plackett-Burman method is comprehensively used for the identification of significant variables as well as their significance levels [27]. From the studied nutrient variables different ingredients have different effects on neomycin production. Ammonium chloride, sodium nitrate, L-histidine, and ammonium nitrate were significant nutritional factors, whereas others were not significant nutritional factors.

Response surface methodology (RSM) is a crucial statistical technique for disclosure interactions among the variables and screening the optimum processes parameters for beneficial responses [28]. As shown in the results above, RSM are powerful tools for identifying the significant nutritional factors and their values for neomycin production. The graphical representations of the regression (3), called the response surfaces plots, were obtained using SAS trial version 9.1, and the interaction effect of variables for neomycin production was presented in Figures 6–9. In the 3-D response surface plot, the yield of neomycin was obtained along with two continuous variables, while the other two variables were fixed constant at their respective zero level (center value of the testing ranges). In all four figures, the maximum predicted value indicated by the surface was confined in the smallest ellipse. Elliptical surfaces are obtained when there is an accurate interaction between the independent variables [21]. The independent variables and maximum predicted values from the figures corresponded with the optimum values of the dependent variables obtained by the equations. The maximal neomycin concentration achieved in this study was 2.7 folds of the neomycin productivity by* Streptomyces fradiae *NCIM 2418. All these suggest that, apart from the method used in this study, another potential alternative for maximizing the neomycin productivity is to design a process with nutrients that stimulate neomycin production.

## 5. Conclusion

Statistical methodology was employed to optimize the neomycin production conditions by* Streptomyces fradiae *NCIM 2418. The Plackett-Burman design and the central composite design were adopted to screen the significant nutritional factors and identify the optimal values for maximum neomycin production. Experimental results indicated that the ammonium chloride, sodium nitrate, L-histidine, and ammonium nitrate had significant effects on neomycin production. However, some significant interactions among the nutritional factors were observed for neomycin production. Under the optimal conditions, the neomycin yield at day 8 reached 19642 g kg^−1^ dry coconut oil cake, which was increased approximately 2.7 folds. This study showed that* Streptomyces fradiae *NCIM 2418 possessed a high neomycin producing capability, which warrants further investigation.

The optimal additional nutrient solution (%) consisted of ammonium chloride 2.00, sodium nitrate 1.50, L-histidine 0.250, and ammonium nitrate 0.250. Under the optimal condition, 20,000 g kg^−1^ dry coconut oil cake could be produced in theoretical experiment and 19642 g kg^−1^ dry coconut oil cake in practical experiment.

## Figures and Tables

**Table 1:** Nutrients and test levels for Plackett-Burman experiment.

Factors	Name	Units	Low level (−1)	High level (+1)
*X*1	Soluble starch	% w/w	0.5	1.0
*X*2	Maltose	% w/w	0.5	1.0
*X*3	Glucose	% w/w	0.5	1.0
*X*4	Copper sulphate	% w/w	0.5	1.0
*X*5	Zinc sulphate	% w/w	0.5	1.0
*X*6	L-asparatic acid	% w/w	0.5	1.0
*X*7	L-glutamic acid	% w/w	0.5	1.0
*X*8	L-Histidine	% w/w	0.5	1.0
*X*9	Sodium Nitrate	% w/w	0.5	1.0
*X*10	Ammonium chloride	% w/w	0.5	1.0
*X*11	Ammonium nitrate	% w/w	0.5	1.0

**Table 2:** Plackett-Burman experimental design for the evaluation of nutritional factors affecting neomycin production by *Streptomyces fradiae *NCIM 2418.

RUN	*X*1	*X*2	*X*3	*X*4	*X*5	*X*6	*X*7	*X*8	*X*9	*X*10	*X*11	*Y* (Neomycin g kg^−1^)
1	1	−1	1	−1	−1	−1	1	1	1	−1	1	2345
2	1	1	−1	1	−1	−1	−1	1	1	1	−1	11896
3	−1	1	1	−1	1	−1	−1	−1	1	1	1	17835
4	1	−1	1	1	−1	1	−1	−1	−1	1	1	8756
5	1	1	−1	1	1	−1	1	−1	−1	−1	1	2453
6	1	1	1	−1	1	1	−1	1	−1	−1	−1	7854
7	−1	1	1	1	−1	1	1	−1	1	−1	−1	14673
8	−1	−1	1	1	1	−1	1	1	−1	1	−1	8676
9	−1	−1	−1	1	1	1	−1	1	1	−1	1	5345
10	1	−1	−1	−1	1	1	1	−1	1	1	−1	16965
11	−1	1	−1	−1	−1	1	1	1	−1	1	1	7453
12	−1	−1	−1	−1	−1	−1	−1	−1	−1	−1	−1	7658

**Table 3:** Coded and actual values of factors in central composite design.

Factor	Name	Units	Levels				
			Axial (−2)	Low (−1)	Center (0)	High (+1)	Axial (+2)
*X*10	Ammonium chloride	% w/w	1.25	1.5	1.75	2.0	2.25
*X*9	Sodium nitrate	% w/w	1.25	1.5	1.75	2.0	2.25
*X*8	L-Histidine	% w/w	0.125	0.25	0.375	0.50	0.625
*X*11	Ammonium nitrate	% w/w	0.125	0.25	0.375	0.50	0.625

**Table 4:** Central composite design arrangement and responses.

RUN	BLOCK	*X*10	*X*9	*X*8	*X*11	*Y* (Neomycin g kg^−1^)
1	1	1.50	1.50	0.250	0.500	18567
2	1	1.50	1.50	0.500	0.250	1765
3	1	1.50	2.00	0.250	0.250	18643
4	1	1.50	2.00	0.500	0.500	18347
5	1	2.00	1.50	0.250	0.250	19642
6	1	2.00	1.50	0.500	0.500	4567
7	1	2.00	2.00	0.250	0.500	10754
8	1	2.00	2.00	0.500	0.250	17643
9	1	1.75	1.75	0.375	0.375	15865
10	1	1.75	1.75	0.375	0.375	15164
11	2	1.50	1.50	0.250	0.250	14678
12	2	1.50	1.50	0.500	0.500	1654
13	2	1.50	2.00	0.250	0.500	17543
14	2	1.50	2.00	0.500	0.250	763
15	2	2.00	1.50	0.250	0.500	765
16	2	2.00	1.50	0.500	0.250	1864
17	2	2.00	2.00	0.250	0.250	11543
18	2	2.00	2.00	0.500	0.500	12543
19	2	1.75	1.75	0.375	0.375	7843
20	2	1.75	1.75	0.375	0.375	12356
21	3	1.25	1.75	0.375	0.375	18532
22	3	2.25	1.75	0.375	0.375	12853
23	3	1.75	1.25	0.375	0.375	456
24	3	1.75	2.25	0.375	0.375	17654
25	3	1.75	1.75	0.125	0.375	15432
26	3	1.75	1.75	0.625	0.375	11754
27	3	1.75	1.75	0.375	0.125	12875
28	3	1.75	1.75	0.375	0.625	18743
29	3	1.75	1.75	0.375	0.375	11754
30	3	1.75	1.75	0.375	0.375	17542

**Table 5:** Effect estimates for the second-order polynomial model.

Term	Estimate	Std. Err.	*t*	Pr > |t|
BLOCK = ‘‘1”	336.2	1200.378	0.280078	0.783824
BLOCK = ‘‘2”	−5604.3	1200.378	−4.66878	0.000439
*X*10	−999.875	547.8951	−1.82494	0.091066
*X*9	3278.0417	547.8951	5.982973	0.0001
*X*8	−2514.375	547.8951	−4.58915	0.000508
*X*11	413.95833	547.8951	0.755543	0.463394
*X*10∗*X*10	95.90625	512.5089	0.187131	0.854447
*X*10∗*X*9	438.3125	671.0317	0.653192	0.525017
*X*10∗*X*8	2550.9375	671.0317	3.801516	0.002201
*X*10∗*X*11	−2645.313	671.0317	−3.94216	0.001686
*X*9∗*X*9	−1563.469	512.5089	−3.05062	0.009289
*X*9∗*X*8	2163.4375	671.0317	3.224047	0.006652
*X*9∗*X*11	1436.9375	671.0317	2.141385	0.051764
*X*8∗*X*8	−428.9688	512.5089	−0.837	0.417715
*X*8∗*X*11	1997.0625	671.0317	2.976108	0.01072
*X*11∗*X*11	125.03125	512.5089	0.243959	0.81107

**Table 6:** ANOVA for response surface quadratic model.

Source	DF	SS	MS	*F*	Pr > *F*
Model	16	1.12	7038	9.76	0.0001
Pure error	3	2717	9059		
Lack of fit	10	6647	6647		
Total	** 29**	** 1.21 **			

DF: degree of freedom; SS: sum of square; MS: mean square.

*R*-square −0.9232; Adj. *R*-square −0.8287.
